# Building a Multi-Sensor Lab for Interactive Communication Research: Challenges, Workarounds, and Lessons Learned

**DOI:** 10.1177/23312165261470622

**Published:** 2026-07-21

**Authors:** Ronny Kurniawan Ibrahim, Kelly Miles, Lisa Maggs, Brooke Luthy, Alan Kan, Michael J. Richardson, Julian Maclaren, Simon Carlile, Zachary M. Smith, Joerg M. Buchholz

**Affiliations:** 1 ECHO Laboratory, Macquarie University Hearing Research Centre, Sydney, NSW, Australia; 2 Performance and Expertise Research Centre, 7788 Macquarie University, Sydney, NSW, Australia; 3 School of Engineering, 7788 Macquarie University, Sydney, NSW, Australia; 4 Google Research, Sydney, NSW, Australia; 5 Cochlear Limited, Sydney, NSW, Australia

**Keywords:** communication research, multi-sensor experiment, device integration

## Abstract

Designing multi-sensor experiments to study interactive communication is costly, complex, and often poorly documented, particularly in terms of implementation challenges and the rationale behind decision-making. Here, we aim to demystify the process by detailing the development of a custom multi-sensor lab designed to monitor comprehensive behavioural and physiological responses during interactive communication. To illustrate the system architecture, we use an implemented paradigm as a worked example: two adults engaged in natural conversation while listening to realistic background noise scenes delivered via open Sennheiser HD-800 headphones. Multi-sensor data were collected using DPA headset microphones, Tobii Pro Glasses 3, a Vicon motion capture system (with Tobii integration), BIOPAC amplifiers (PPG, EDA, ECG, respiration, temperature), a Fitbit Sense 2 smartwatch, and six Logitech BRIO 4K ultra HD video cameras recording via Open Broadcaster Software (OBS). Due to high bandwidth demands and varying sampling rates (1 Hz to 48 kHz) among the different devices, all systems recorded independently which posed a significant synchronisation challenge. A central RME soundcard delivered trigger pulses to the Tobii, BIOPAC, OBS, and Vicon systems to mark stimulus onset/offset. Redundant synchronisation mechanisms were implemented to mitigate the risk of trigger failure and bespoke processing pipelines were developed to synchronise data streams. We share key challenges and creative solutions encountered in building such a lab, to help researchers understand and anticipate common pain points and make more informed decisions when implementing their own multi-sensor setups.

## Introduction

Human communication is inherently interactive and multi-modal. It unfolds through rapid, reciprocal exchanges in which talk, gesture, facial expression, gaze, and body position are tightly coupled between partners (Barnes & Bloch, 2019). Yet, despite major advances—predominantly driven by the development of improved hearing devices and algorithms—most hearing research has historically been conducted in tightly controlled laboratory settings, relying on single-talker or single-listener paradigms coupled with simplified auditory scenes. Although such approaches provide valuable experimental control, they do not fully capture the reciprocal, embodied, and context-dependent nature of interactive conversation in which people with hearing loss, and the devices that support them, must ultimately function. At the same time, the methodological landscape is broader than a simple contrast between tightly controlled laboratory studies and unconstrained real-world observation. A growing body of work has used ecological momentary assessment (EMA) to characterise hearing-related experiences in everyday life (Burke & Naylor, 2022; Holube et al., 2020), while other studies have employed virtual and simulated environments to retain experimental control while approximating real-world communicative demands (Bak et al., 2025; Hohmann et al., 2020; Sonible, 2020). Together, these approaches reflect a broader shift in the field toward methods that better represent how communication unfolds in everyday life, with increasing emphasis on embodied, multimodal, and dynamically coordinated processes that support real-world interaction.

A substantial body of research has examined communication as it unfolds in natural settings using conversation-analytic, ethnographic, and interactional approaches. This work has provided detailed accounts of turn-taking, repair, gesture, gaze, and partner coordination during real-world interaction across homes, schools, restaurants, and clinical environments, including among people with hearing loss (e.g., Caissie & Rockwell, 1994; Church et al., 2017; Lind et al., 2004; McKellin et al., 2007; Pajo & Laakso, 2023; Tye-Murray, 1991). However, because these studies typically prioritise naturally occurring interaction, they are often conducted in acoustically uncontrolled environments, where background noise is part of the communicative context rather than an experimentally manipulable variable. As a result, they provide rich insight into real-world communication in noise, but offer limited experimental control and reproducibility.

Other approaches, including ecological momentary assessment and field-based sensing, have characterised the lived experience of listening and communication in everyday environments, often using mobile or wearable technologies such as smartphones, smartwatches, and hearing devices to capture self-report, contextual, and physiological data (Andersson et al., 2023, 2025; Burke & Naylor, 2022; Holube et al., 2020). While these approaches provide valuable insight into real-world listening and communication experiences, they are typically not designed to quantify fine-grained, moment-to-moment behavioural adjustments and coordination between conversation partners.

More recently, studies of interactive communication that specifically focus on background noise have shifted aspects of real-world interaction into controlled laboratory environments, enabling systematic manipulation of acoustic conditions alongside synchronised measurement of speech, gaze, movement, and other behavioural signals. These studies demonstrate the value of capturing communication as a coordinated multi-sensory process and show that acoustic conditions can systematically shape speech levels, utterance timing, gaze, movement, and conversational coordination (Beechey et al., 2018; Hadley et al., 2021; Miles et al., 2023; Petersen, 2024; Petersen et al., 2022; Slomianka et al., 2025; Sørensen et al., 2021, 2024; Weisser & Buchholz, 2019). Related work has also begun to articulate experimental design considerations for interactive conversation studies, including small-group paradigms and the trade-offs involved in balancing ecological validity with experimental and statistical control (Nicoras et al., 2025). As a result, a methodological space is emerging between richly described real-world interaction and experimentally tractable, synchronised, multi-sensor datasets capable of supporting systematic manipulation and replication.

The present paper contributes to this space by focusing on a practical issue that remains technically demanding and under-reported: how to build, integrate, troubleshoot, and operate a multi-sensor laboratory for interactive communication research. Method sections often condense months of engineering work into a single sentence, leaving other groups to reconstruct complex solutions through extensive trial and error. This lack of transparent, implementation-focused guidance slows progress and limits reproducibility across the field.

Here, we provide a systematic framework for addressing these challenges by transparently documenting the design, implementation, and integration of a custom-built multi-sensor communication laboratory. Our approach combines realistic auditory scene presentation with simultaneous capture of speech, gaze, head and body movement, and autonomic physiology to create a unified dataset reflecting behavioural and physiological responses during interactive conversation. To investigate measurement beyond the laboratory, we also incorporated wearable sensors (using a smartwatch), capable of recording cardiovascular, electrodermal, thermal, and movement signals in naturalistic environments.

To support the methodological demands of interactive communication research, we decomposed the laboratory into a series of functional subsystems and decision layers that together define the overall architecture. In the following sections, we describe our implementation of each component subsystem first, followed by a discussion on key design choices and considerations. While the example implementation described here focuses on dyadic conversation, the architecture is intentionally modular and scalable, such that it can, in principle, be extended to capture group conversations of arbitrary size by replicating and integrating additional sensing nodes. Together, these design principles and practical solutions aim to support researchers in developing scalable, synchronised, and ecologically-grounded platforms for advancing hearing research in controlled, realistic communicative contexts.

## Physical Environment

### Our System

**Figure 1.**
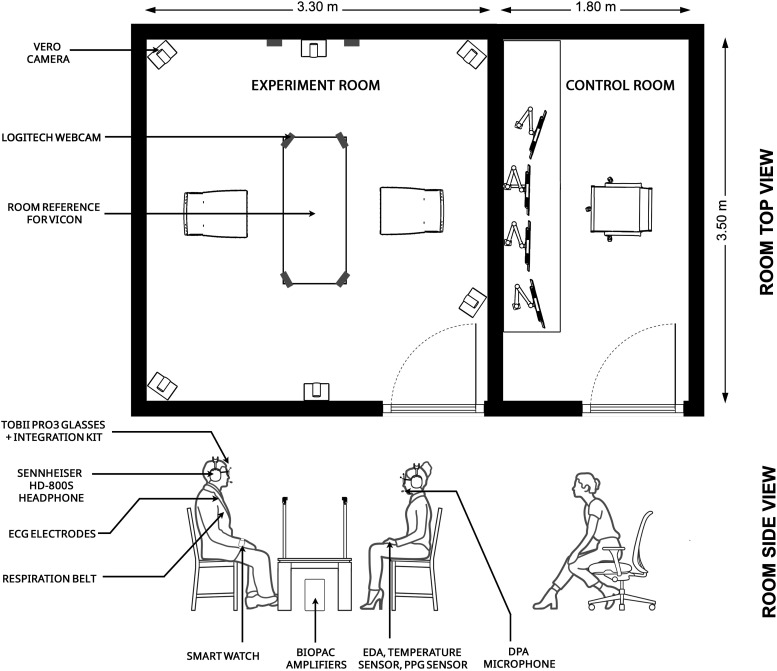
Schematic of the multi-sensor laboratory setup shown in top and side views. The top view illustrates the spatial layout of the experiment room (3.30 m × 3.50 m) and adjacent control room (1.80 m × 3.50 m), with two participants seated face-to-face across a central table. The experiment room is equipped with Vicon Vero cameras, a Logitech webcam, and room reference markers for motion capture, video recording, and spatial calibration, while the control room houses the experimenter workstation. The side view shows participant positioning and instrumentation, including Tobii Pro Glasses 3, Sennheiser HD-800 headphones, DPA microphones, ECG electrodes, a respiration belt, BIOPAC amplifiers, a smartwatch, and sensors for electrodermal activity (EDA), temperature, and photoplethysmography (PPG). This setup enables synchronised recording of conversational behaviour, audio, eye tracking, motion capture, and physiological responses during face-to-face interaction

The laboratory space was set up in an acoustically treated and shielded room (3.3 m x 3.5 m) to minimise external noise interference. Room lighting was kept constant across sessions to support accurate and consistent video capture and gaze tracking. A separate control room (1.8 mx 3.5 m) housed the experimenter and acquisition computers, enabling real-time monitoring of all subsystems without influencing participant behaviour (see Figure 1). An intercom system, consisting of a wall-mounted microphone and loudspeaker in the experimental room, and a headset (microphone and headphones) in the control room, was installed to enable communication during the experiment session.

Within the Experiment room, two participants were seated face-to-face at a distance of approximately 145 cm (head-to-head), separated by a coffee table measuring 60 cm in width and 120 cm in length (Figure 1, room side view). Both individuals sat on fixed-height chairs (seat height: 51 cm; backrest height: 95 cm) that remained in fixed floor positions throughout the session. While remaining seated, participants were free to engage in conversation naturally, which included shifting posture, gesturing, and turning their heads.

### Key Considerations

In each section below we identify key design factors that directly constrained system performance, data quality, participant safety or interpretability of the resulting multimodal data. These are not intended to be exhaustive descriptions of all possible implementation choices, but rather the factors that were most consequential in our system and are likely to generalise to other laboratories attempting similar multi-sensor recordings.

In setting up a multi-sensor laboratory, several key considerations are needed regarding the physical space and environmental conditions. These are essential for ensuring consistent sensor visibility and reliable recording quality across multi-sensor data streams as well as across experimental sessions. Here, we list these considerations and provide a set of recommended parameters using a worked-example paradigm. Empirical findings from this paradigm are reported separately in this special issue (Luthy et al., 2026). For the physical environment, we focus on room acoustics, lighting and spatial stability because these factors directly affect the reliability and interpretability of the data.

**Room acoustics and noise:** The experiment room should exhibit controlled acoustic properties appropriate for conversational speech research. At a minimum, laboratories should quantify the mid-frequency reverberation time (RT60; typically 500–2000 Hz) and the ambient background noise level (A-weighted, dBA SPL) with all equipment powered but idle. In the worked-example paradigm, testing took place in a room measuring 3.5 × 3.3 × 2.4 m, with a reverberation time of T30 = 0.09 s and an ambient background noise level of less than 26 dBA SPL. These room-acoustic characteristics directly affect the communicative demands placed on participants and, consequently, the behavioural and physiological measures obtained. Elevated background noise has been shown to increase vocal effort and degrade speech intelligibility, while also altering conversational dynamics such as the timing and variability of turn exchange (Bottalico et al., 2022). Reverberation likewise reduces speech intelligibility in realistic listening situations, and in interactive tasks, both noise and reverberation have been shown to influence utterance structure, head movement, and broader patterns of conversational behaviour (Archer-Boyd & Martin, 2023; Guastamacchia et al., 2024). Related work further indicates that noisy environments elicit systematic adaptations in speech level, utterance duration, interpersonal movement, gaze, and listener strategy, demonstrating that room acoustics can shape multi-sensor outcome measures well beyond the speech signal alone (Hadley et al., 2019; Miles et al., 2023). For these reasons, reverberation and noise floor should be treated as core experimental variables rather than incidental background conditions. Their careful measurement and reporting are critical for interpreting findings within a study and for supporting meaningful comparison across studies. Guidance on recommended maximum background noise levels is, for example, provided in ITU-T P.800, and standardised methods for measuring room acoustic parameters, including reverberation, are described in ISO 338-1 (International Telecommunication Union, Telecommunication Standardization Sector, 1996).

**Lighting conditions:** Lighting should remain stable and consistent across recording sessions to support reliable video capture, gaze tracking and computer-vision-based analyses. We recommend an illumination level of approximately 950 lux at a correlated colour temperature of 4000 K, while also considering whether the lighting environment is artificial, natural or mixed.

This recommendation was guided by practical lighting standards from television and studio production, where typical illumination levels range from approximately 800 to 2000 lux (Department of the Environment and Energy, 2019; European Broadcasting Union, 2017; Free TV Australia, 2019; Strand Lighting, 1986), and by standard office lighting recommendations, which commonly fall between 300 and 1000 lux (Free TV Australia, 2019; University of Toronto, Environmental Health and Safety, 2023). The selected level was intended to minimise glare, reduce thermal build-up, maintain participant comfort, and preserve high-quality video recording. In addition, lighting should be positioned above and slightly in front of the participant to minimise periocular shadowing, which can reduce the reliability of eye-tracking and computer-vision-based facial landmark detection.

**Spatial stability:** Furniture placement, chair height, and participant positioning should be fixed across sessions to maintain consistent sensor geometry, camera fields of view, and tracking accuracy. Any deviations from the standard setup should be documented, as even small spatial changes can propagate into systematic errors in gaze, motion-capture, and pose-estimation data. In our system, the spacing of the chairs and the inclusion of a centrally-placed table were chosen to support unobstructed sight lines for all camera views.

## System Architecture

### Our System

**Figure 2.**
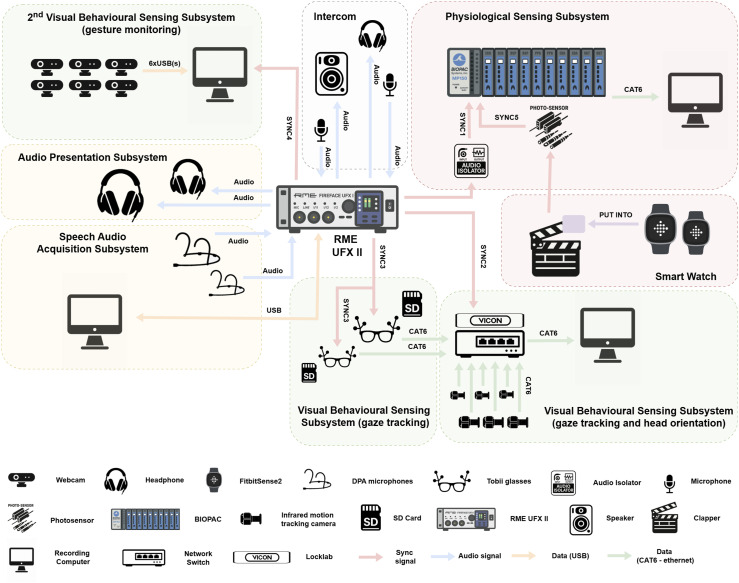
Schematic of the multi-sensor data acquisition, interaction, and synchronisation framework used in the multi-sensor laboratory. The RME Fireface UFX II served as the central hub linking the major subsystems, including audio playback, speech recording, and intercom communication, while also providing synchronisation signals to the other subsystems. Smartwatches were placed in an enclosure which was mounted on the clapper arm. (Colour coding note: Lines: audio signals (blue); data via USB (orange); data via ethernet cable (CAT) (green); synchronisation signals (red); watches placed into enclosure (purple); Rectangle dotted lines: Physiological Sensing Subsystems (red); Visual Behavioural Sensing Subsystem (green); Audio (orange))

In our example experiment, we were interested in understanding how dyadic conversation and interactions naturally change in different real-world environments with varying levels of background noise. In particular, we were interested in changes in speech levels, head and gaze behaviour, gesturing, and biophysical signals. Hence, we needed a system capable of creating reproducible acoustic scenes while recording all the parameters of interest during the dyadic conversation. Our implemented system is shown in Figure 2.

Our system is organised as four interconnected subsystems, each responsible for a distinct class of signals. Rather than functioning as isolated measurement tools, these subsystems operate as coordinated components within a distributed architecture, designed to capture the behavioural, acoustic, and physiological dimensions of natural conversation in parallel. This subsystem-based architecture reflects a signal-driven design approach, in which each component is organised according to the type of information it captures or delivers. At the same time, all subsystems remain temporally and spatially aligned within the broader system. Together, these subsystems support the recording of conversational speech, auditory context, embodied behaviour, and autonomic state within a unified experimental framework.

The four core subsystems are:

**Auditory Presentation Subsystem** Responsible for the controlled delivery of auditory scenes, including background noise, interfering talkers and other spatialised environmental sounds. This subsystem controls stimulus timing, intensity, and acoustic structure, ensuring reproducible playback conditions across experimental sessions. Synchronisation triggers are sent from the auditory presentation subsystem to the other acquisition subsystems.

**Speech Audio Acquisition Subsystem** Captures high-fidelity conversational speech from each participant, enabling detailed analysis of vocal output, turn-taking behaviour, and speech modifications in response to listening demands.

**Visual Behavioural Sensing Subsystem** Captures gaze, head orientation, body posture, gesture, and video-based recordings of interaction. This subsystem comprises two complementary components: eye/head tracking using Tobii Pro Glasses 3 integrated with Vicon, and multi-camera video recording for gesture monitoring, behavioural annotation, and pose estimation.

**Physiological Sensing Subsystem** Monitors autonomic nervous system responses, including cardiovascular activity, electrodermal responses, respiration, and skin temperature, providing insight into listening effort, stress, and physiological load during conversation.

Although each subsystem operates on an independent acquisition platform with its own sampling characteristics and technical constraints, they are unified through a centralised synchronisation framework and a shared experimental timeline. This approach enables simultaneous measurement of multiple interacting systems while maintaining the flexibility needed to accommodate high-bandwidth data streams and complex sensor configurations.

The following sections describe each subsystem in detail, outlining the technical implementation, design rationale, and integration within the broader multi-sensor architecture. This is followed by a description of how data synchronisation was handled for the entire system.

### Auditory Presentation Subsystem

The auditory presentation subsystem was responsible for reproducing real-world acoustic scenes that were played to each conversation participant through Sennheiser HD-800 open-back headphones. Audio signal processing was implemented in MATLAB 2022a, while real-time scene presentation was implemented using the sfplay object in Max/MSP (Cycling ’74; Version 8.6.5) in conjunction with an RME UFX II audio interface. The Max/MSP patch is available on GitHub and can be provided by the authors upon request. All audio stimuli were rendered at a sampling rate of 48 kHz with 24-bit resolution to ensure high-fidelity playback.

Realistic noise environments were selected to reflect a range of everyday scenarios and were drawn from the Ambisonic Recordings of Typical Environments (ARTE) database (Weisser & Buchholz, 2019), with an additional “party” environment featuring background music incorporated from Weisser et al. (2021). These recordings provided diverse auditory contexts capturing common sources of communicative interference in natural acoustic environments. The background noise environments are summarised in Table 1, which includes unweighted free-field RMS levels calculated across their entire stimulus duration. Note that these values differed slightly from their nominal (real-world) values to provide a more equally spaced distribution as well as to avoid uncomfortable loudness in the Party environment (with a real-world level of 95 dB SPL).

**Table 1.** Background Noise Environment Details

Environment	dB SPL	Description
Office	60	Open plan office, people typing, chatting, and talking on the phone
Cafe	70	Indoor (company) cafe at medium occupancy before lunch, recorded next to a wall
Food court	80	Very noisy food court in a shopping mall during lunch
Party	87.5	A party in a reverberant room with lots of people talking and music playing

All scenes were converted into non-individualised binaural recordings using the 31-channel higher-order Ambisonics signals provided by ARTE and the head-related transfer functions provided by Oreinos and Buchholz (2013), ensuring accurate spatial rendering of the original Ambisonic sound fields as well as free-field equivalent ear levels (i.e., in-ear levels as measured inside the original sound field). The resulting binaural signals were simultaneously presented to both participants over open-back headphones without head-tracking capabilities, using the two separate headphone outputs of the RME UFX II sound card and mirrored by the RME mixer. Equalisation filters were applied to flatten the frequency response of the headphones when measured on a Bruel&Kjaer Type 5128-C Head and Torso Simulator (HATS). To account for the acoustic transmission characteristics of the headphones, the binaural signals were additionally low-pass filtered with a 1^st^-order Butterworth lowpass filter with a cut-off frequency of 3,000 Hz. This filtering was applied after noise stimulus calibration and replicated the slight high-frequency attenuation observed when measuring an external (frontal) talker through the headphones using a Bruel&Kjaer Type 5128-C HATS (Weisser & Buchholz, 2019). Each noise file had a duration of 2.5 minutes and was looped as needed to accommodate longer stimulus presentations. Headphone presentation was selected to minimise background-noise spill into the recorded speech signals, thereby improving signal quality and facilitating more reliable analysis of participant speech.

Note that the binaural headphone reproduction did not incorporate head tracking to avoid the associated technical effort and cost. Consequently, listeners’ head movements would have caused the simulated auditory scene to rotate with their head rather than remain spatially fixed in the room. This limited the ecological validity of our binaural reproduction as head motion does not only improve the authenticity of the auralized environments but also their externalisation (Hendrickx et al., 2017) and the utilisation of head-shadow effects to improve the signal-to-noise ratio at the participant’s ears (Grange et al., 2018). Future work should therefore aim to integrate real-time head tracking with the audio presentation system to support stable and ecologically valid spatial reproduction.

### Speech Audio Acquisition Subsystem

The speech audio acquisition subsystem was responsible for recording synchronised, high-fidelity conversational speech while minimising acoustic distortions. For this purpose, directional headset microphones (DPA d:fine™ 4088) were employed to maximise participant-specific speech capture and minimise acoustic crosstalk between the two talkers as well as room reverberation. Potential plosive and proximity-related artefacts were minimised during acquisition through careful microphone positioning as well as the use of foam filters. Any remaining artefacts were removed during post-processing using the de-plosive and de-bleed functions in iZotope RX software. Audio was captured at a sampling rate of 48 kHz with 24-bit resolution, providing a broad dynamic range suitable for subsequent analyses. Nevertheless, the microphone preamplifier gains provided by the RME sound card still had to be carefully set to accurately capture the large range of vocal effort levels elicited by the different background noise conditions, particularly to avoid clipping during the loudest test conditions. Audio recording was implemented in Max/MSP using the sfrecord object to record speech from two participants.

In addition to the two speech channels, the recording included recording the two-channel noise playback and a dedicated synchronisation trigger channel. In total, five audio channels were recorded: speech (Participant 1), speech (Participant 2), noise (left), noise (right), and synchronisation.

### Visual Behavioural Sensing Subsystem

The visual behavioural sensing subsystem was responsible for recording synchronised eye gaze, head, positional and gestural data from participants. To ensure high-fidelity capture of this multi-modal data, two separate recording systems were employed.

The first system targeted the capture of eye gaze, head and positional data (see bottom right of Figure 2). Two pairs of Tobii Pro Glasses 3 were integrated with a Vicon motion-capture system consisting of six Vicon Vero cameras, a LockLab unit, and a gigabit network switch. Cameras were mounted at a height of approximately 2.2 m and distributed around the experiment room to maximise coverage of the interaction space. The global coordinate system was defined with its origin at the midpoint of the table separating the two participants (see Figure 1, room top view), providing a stable spatial reference for dyadic interaction.

Eye gaze behaviour was recorded using Tobii Pro Glasses 3, which are equipped with inward-facing cameras mounted on the lenses to capture binocular eye movements. Data acquisition was managed through the Tobii proprietary software (Version 3-1.18.6), with recordings stored locally on an integrated SD card and simultaneously streamed to the Vicon Nexus software (Version 2.16) for real-time monitoring and synchronisation with head-tracking data. Gaze data were additionally recorded via the Vicon Nexus software to support integrated behavioural analysis.

Data recorded to the SD card included: (1) the position of the left and right eyes, (2) gaze vectors for each eye, (3) a combined gaze vector representing the intersection of left and right gaze directions, (4) pupil diameter, and (5) synchronisation input and output signals for alignment with other datastreams.

This configuration enabled precise capture of gaze dynamics, pupil-linked arousal, and temporal alignment with additional physiological and behavioural measures. The Tobii glasses also record video from a forward-facing scene camera located between the lenses. Scene video was recorded at a resolution of 1920 × 1080 pixels at 25 fps. Audio captured by the scene camera was retained as an additional redundant synchronisation signal.

Head movements were recorded by the Vicon motion-capture system. A unique constellation of six reflective markers (Tobii-Vicon integration kit) were attached to each pair of Tobii glasses which enabled individual tracking of the location of the Tobii scene camera within the room, which served as the reference point for head tracking. Captured data included three-dimensional head position and orientation.

Output parameters comprised translational position (X, Y, Z) and rotational orientation expressed as pitch, yaw, and roll. These measures enabled continuous quantification of head movement, orientation and relative distance of the two participants during interactive communication, and supported alignment with gaze, speech, and physiological data streams.

The second component of the Visual Behavioural Sensing Subsystem targeted the capture of body posture and hand gestures (see top left of Figure 2). This subsystem provided complementary visual documentation of the interaction from multiple camera perspectives, supporting qualitative interpretation, behavioural annotation, and verification of synchronisation across data streams. This system comprised of six Logitech BRIO webcams.

Four cameras were positioned approximately 90 cm above the floor at the corners of the coffee table (see Figure 1), always two cameras capturing one of the individual participants. Two additional cameras were mounted on the side wall to capture wider-angle views of the interaction between the two participants during communication. Video footage from the cameras were recorded using the Open Broadcaster Software (OBS Version 30.0.0).

Within OBS, a recording canvas with a resolution of 5760 × 2160 pixels was configured to capture the six full-HD video streams simultaneously at 60 fps. Camera parameters, including focus, white balance, and exposure, were standardised across devices using the WebCameraConfig application to ensure consistent visual characteristics across recordings. Audio from each webcam was also recorded in OBS and mapped to separate channels.

The resulting output consisted of a single 5760 × 2160 video file at 60 fps, encoded using the H.264 codec, with six corresponding audio channels. These audio recordings served as redundant synchronisation cues for alignment across video streams and with other modalities. During post-processing, the composite video was segmented into six individual video streams corresponding to each camera view. These streams were subsequently used for conversation analysis and automated pose estimation, implemented using OpenPose (Cao et al., 2019).

### Physiological Sensing Subsystem

The physiological sensing subsystem was designed to record autonomic and peripheral physiological responses during interactive communication. Laboratory-grade physiological data were collected using BIOPAC amplifiers, including electrocardiography, electrodermal activity, respiration, photoplethysmography, and peripheral skin temperature. In parallel, consumer-grade wearable sensors were implemented using a Fitbit Sense 2 smartwatch, which enabled collection of complementary physiological and movement-related signals. Together, these recordings provided measures of cardiovascular, electrodermal, respiratory, thermal, and movement-related responses during the conversation task.

The laboratory-grade system was a BIOPAC acquisition system (BIOPAC Systems Inc., Goleta, CA) comprising of a data acquisition module (MP150), a universal interface module (UIM100C), and a set of specialised bioamplifier modules with corresponding transducers. This configuration enabled the simultaneous acquisition of multiple physiological signals from the two test participants. The universal interface module was used to receive external synchronisation trigger signals from the auditory playback subsystem and from a photosensor mounted behind the clapper’s arm (see Figure 2: SYNC1 and SYNC5).

Electrocardiographic (ECG) activity was measured using an ECG100C amplifier with two surface electrodes positioned on the left and right sides of the chest. Photoplethysmography (PPG) was recorded using a PPG100C amplifier connected to a TSD200 transducer, providing an index of peripheral blood volume changes. Respiratory activity was assessed using an RSP100C amplifier in conjunction with a TSD201 respiration transducer, which detects thoracic expansion and contraction; the sensor was placed on the chest beneath the sternum.

**Figure 3.**
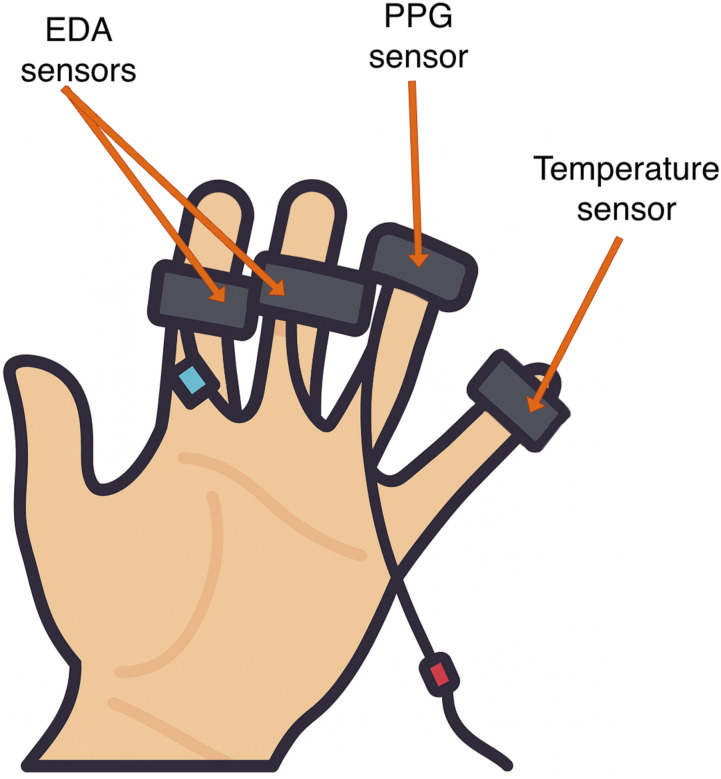
Schematic illustration of the placement of BIOPAC peripheral sensors on the non-dominant hand. Electrodermal activity (EDA) sensors were positioned on the index and middle fingers, the photoplethysmography (PPG) sensor was placed on the ring finger, and the temperature sensor was attached to the little finger

Skin temperature was measured using an SKT100C amplifier with a TSD202 transducer positioned on the third knuckle of the little finger. Electrodermal activity (EDA) was acquired using a GSR100C amplifier with a TSD203 transducer attached to the second knuckles of the index and middle fingers. The PPG, EDA, and skin temperature sensors were all placed on the palmar surface of the non-dominant hand (see Figure 3).

All signals were acquired simultaneously and sampled at 2 kHz to facilitate accurate multi-sensor synchronisation with other behavioural and physiological measures. Data acquisition was performed using AcqKnowledge software (Version 5.0.8.1, BIOPAC Systems Inc.) to record signals from the bioamplifiers. In total, twelve data streams were recorded, comprising two trigger channels (one from noise playback and one from the photosensor), two ECG channels, two PPG channels, two EDA channels, two respiration channels, and two skin temperature channels.

In addition to laboratory-grade sensors, a consumer-grade wearable device was incorporated into the physiological sensing subsystem to evaluate the feasibility of a transportable system suitable for ecological momentary assessment (EMA). For this study, we used two Fitbit Sense 2 smartwatches (Fitbit Inc., San Francisco, CA), which integrates multiple sensing modalities within a single wearable platform. Data acquisition was facilitated through a custom Google-developed wearable application that enabled access to raw physiological and motion signals at higher sampling rates than those typically available via standard consumer interfaces. There are alternative options available for continuous physiological monitoring in real-world settings, such as the Empatica EmbracePlus (Empatica, 2026).

The application recorded data from the photoplethysmography (PPG) sensor, electrodermal activity (EDA) sensor, skin temperature sensor, and inertial measurement unit (IMU) of the smartwatch. PPG signals were sampled at 20 Hz, enabling extraction of cardiovascular metrics such as heart rate and heart rate variability. EDA was sampled at 20 Hz, providing indices of sympathetic arousal, while skin temperature was sampled at 1 Hz to capture slower fluctuations in peripheral temperature. IMU signals were recorded at 100 Hz, providing high-resolution measures of linear acceleration and angular velocity suitable for fine-grained analyses of movement and gesture which can be compared to the data obtained from the visual behavioural sensing subsystem.

## Data Synchronisation

Precise temporal synchronisation is essential in multi-modal hardware setups, where heterogeneous data streams must be integrated into a coherent temporal framework for analysis. Physiological, behavioral, and audio-visual recording systems typically operate on independent acquisition platforms with different sampling rates, latencies, and internal clocks, creating significant challenges for temporal alignment — challenges that are well documented in distributed sensing and data-fusion contexts, where unsynchronised streams can lead to temporal drift, mis-time-locked events, and spurious correlations that compromise data integrity (Bennett et al., 2017; Wåhslén, 2013; Yiǧitler et al., 2020).

In the present system, sampling rates for each subsystem were selected according to device constraints and integration requirements (see Table 2). Where adjustment was not possible, manufacturer-default settings were retained (e.g., the smartwatch). Where subsystems could be aligned, sampling rates were matched accordingly — for example, audio presentation and acquisition were both configured at 48 kHz to correspond with the video audio. Where higher temporal resolution was both desirable and technically feasible, the maximum supported sampling rate was used, such as 2 kHz for the BIOPAC physiological recordings. As illustrated in Figure 2, four computers were used to realise the different data acquisition subsystems. These were:

1. Audio Playback/Recording computer: For the Audio Presentation and Speech Audio Acquisition Subsystems.
2. Vicon/Tobii computer: For the 1^st^ Visual Behavioural Sensing Subsystem (gaze tracking and head orientation).
3. Webcam/OBS computer: For the 2^nd^ Visual Behavioural Sensing Subsystem (gesture monitoring).
4. ANS/BIOPAC computer: For the Physiological Sensing Subsystem.

**Table 2.** Overview of Hardware and System Requirements

Subsystem	Requirement	Hardware	Sampling rate	Acquired/presented data	No. of datastreams	Software
Speech Audio Acquisition	Speech	DPA close mic; RME UFX II	48 kHz	Speech (2), noise files, SYNC (1, 2, 3, 4), timecode	5	Max/MSP; MATLAB
Visual Behavioural Sensing	Head movement	Vero cameras (6); Locklab; gigabit switch; Tobii Pro Glasses3 constellation	50 Hz	Position [X, Y, Z]; orientation [pitch, yaw, roll]	6 (2)	Nexus
Visual Behavioural Sensing	Gaze movement	Tobii Pro Glasses3	50 Hz	Position [L,R][X,Y,Z]; gaze [L,R][X,Y,Z]; combined gaze [X,Y,Z]; pupil size [L,R]	17 (2)	Tobii software
Visual Behavioural Sensing	Gesture monitoring	Logitech BRIO webcams (6)	60 fps	Video footage	6	OBS
Physiological Sensing	Heart rate	BIOPAC ECG100C; PPG100C	2 kHz	ECG and PPG waveforms	2 (2)	AcqKnowledge
Physiological Sensing	Respiration	BIOPAC RSP100C	2 kHz	Respiration waveform	1 (2)	AcqKnowledge
Physiological Sensing	Electrodermal activity	BIOPAC EDA100C	2 kHz	Galvanic skin response (finger)	1 (2)	AcqKnowledge
Physiological Sensing	Temperature	BIOPAC SKT100C	2 kHz	Skin temperature (finger)	1 (2)	AcqKnowledge
Physiological Sensing	smartwatch	Fitbit Sense 2	1–100 Hz	PPG (wrist), galvanic skin response (wrist), temperature (dorsal wrist), acceleration (wrist), angular velocity (wrist), and timecode	15 (2)	Custom app
Audio Presentation	Noise playback	RME UFX II; Sennheiser HD-800	48 kHz	24-bit WAV files of different noise environments	2	Max/MSP; MATLAB

*Note.* The number in parentheses indicates the number of devices or participants.

Beyond these four computers, the smartwatches themselves recorded data which were downloaded from the smartwatches at the end of each session.

Given the distributed nature of the data acquisition hardware, we needed to ensure that the data acquired could be reliably synchronised during post-processing. We therefore implemented a distributed acquisition architecture with centralised temporal coordination, whereby explicit synchronisation signals were transmitted to each data-acquisition unit.

The audio playback/recording computer equipped with a RME UFX II USB interface was selected as the central synchronisation unit because it was operated at the highest sampling rate and had the ability to generate arbitrary waveforms with configurable amplitude and frequency characteristics to match the trigger signals expected by the other acquisition units. From this central unit, hardware trigger signals were distributed to all acquisition systems to mark stimulus onsets (start of noise playback), stimulus offsets (end of noise playback), and test condition (block) transitions. These triggers were recorded by the different acquisition units and served as reliable, low-latency temporal anchors for cross-system alignment.

However, not all data acquisition units readily accepted trigger signals from the RME interface — notably the Fitbit smartwatches and the webcam recording system — and initial testing revealed that synchronisation signals were occasionally missed by systems operating at lower sampling rates. Given the complexity and distributed nature of our acquisition architecture, redundancy was therefore intentionally incorporated into the synchronisation strategy to safeguard against trigger failures and support robust post-hoc temporal alignment. This is particularly important in our high-bandwidth multi-sensor settings, where a single-point synchronisation failure can compromise an entire dataset.

**Figure 4.**
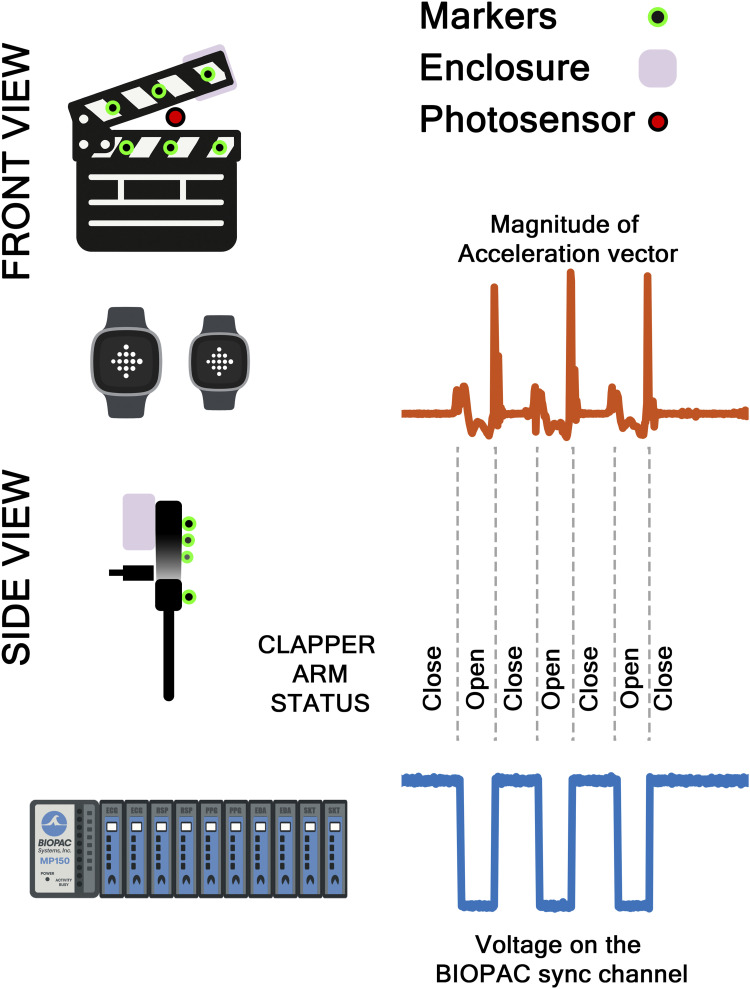
Schematic of the custom synchronisation clapper in front and side views. Six motion-tracking markers were mounted on the arm and body to enable redundant motion-capture-based synchronisation. A small enclosure attached to the clapper arm housed the smartwatch, while an external photosensor connected to the BIOPAC detected whether the arm was open or closed. The peak acceleration at clapper impact provided a timing marker for the watch. This enabled temporal alignment of the smartwatch and BIOPAC physiological recordings during acquisition

To address this, a clapperboard (referred to hereafter as a “clapper”) was used as an additional synchronisation mechanism across all data acquisition systems, providing either a primary or backup trigger signal depending on the subsystem (see Figure 4). The clapper created a temporal marker that was picked up by all microphones (headsets, Tobii glasses, Webcams), the webcams of the OBS video recording system, and the scene camera on the Tobii glasses worn by the participants. It produced a distinctive high-amplitude audio transient aligned with a visible motion cue, enabling reliable cross-modal alignment between audio and video streams during post-processing. This provided the primary synchronisation cue for the Webcam OBS recordings with reference to the speech recordings of the audio playback and recording system, the central synchronisation unit.

For the clapper to be recorded by the Vicon/Tobii system, six reflective markers were affixed to the clapper body and arm (see Figure 4). The rapid closing motion of the clapper produced a clearly identifiable event in the Vicon kinematic data stream, enabling precise alignment of motion-capture data with the audio and video-based timing cues. This provided a secondary synchronisation signal to the Vicon system in the event of RME trigger failure.

For the clapper to be recorded by the BIOPAC system, a photosensor was placed behind the clapperboard’s arm (Figure 4) and connected to an analogue input channel of the BIOPAC system (Figure 2). The photosensor utilised a paired light transmitter and receiver to track the opening and closing of the clapper. When the clapper was closed, the emitted light was reflected to the sensor, producing a high-voltage output. Opening the clapper interrupted the reflected light path, resulting in a low-voltage output. This provided a secondary synchronisation signal to the BIOPAC system in the event of RME trigger failure.

As the smartwatches did not support the recording of external trigger signals, synchronisation was inferred from the onboard accelerometry signals. For this purpose, the smartwatches were placed within a small rigid enclosure attached to the clapper arm. Closing the clapper generated a mechanical impact upon contact between the arm and the clapper body, producing a prominent transient in the acceleration data. The magnitude of the acceleration vector was used as the synchronisation measure, providing a robust synchronisation marker insensitive to device orientation within the enclosure.

Finally, the loudspeaker of the intercom system that was connected to the RME interface (Figure 2) was used to deliver a 0.5 s, 1 kHz sine-tone burst at the start and end of each test condition. This tone was reliably captured by the microphones of the main speech recording system, the Tobii glasses, and the webcams of the OBS recording system, and served as a secondary fallback mechanism when primary synchronisation pulses distributed from the RME to the Tobii glasses or OBS system were not detected.

Table 3 summarises all synchronisation trigger signals generated by the Audio Playback and Recording computer with the RME interface, including their timing parameters and routing paths used to mark the beginning and end of each experimental test condition. The trigger signals were pre-filtered to boost the frequencies below 5Hz as the UFX II applies a high-pass filter at its outputs. Note that some sound cards provide fully DC-coupled audio channels, eliminating the need for high-pass compensation filters.

**Table 3.** Synchronisation Details

Synchronisation purpose	Source	Destination	Details
Noise playback and BIOPAC	RME	UIM100C BIOPAC	0.5 s, 2 V V_pp_ square wave at 20 Hz
Noise playback and OBS	RME	OBS PC	0.5 s, 5 V V_pp_ square wave at 20 Hz
Noise playback and Vicon	RME	Locklab	0.5 s, 5 V V_pp_ square wave at 20 Hz
Noise playback and Tobii Glasses	RME	Tobii Controller Unit	0.05 s, 3.3 V V_pp_ square wave at 20 Hz
Noise playback, Tobii Glasses, OBS, and Max/MSP	RME	Max/MSP, OBS, and Tobii Controller	0.5 s, 1 kHz sine tone
Smartwatch and BIOPAC	Clapper and photosensor	UIM100C BIOPAC	Three claps
Smartwatch and Vicon	Clapper	Vicon PC	Three claps
Multicamera visual timing synchronisation	Clapper	OBS PC	Three claps
Multicamera audio–visual timing synchronisation	Clapper	OBS PC and Tobii Controller	Three claps

### Key Considerations

In implementing the data acquisition system described above, a number of challenges influenced design decisions. In this section, we discuss each of these challenges and outline the practical considerations relevant to system design and implementation.

### Data Acquisition (Continuous vs Segmented Recording)

One of the central design considerations in a multi-sensor recording system is whether to adopt a continuous recording strategy or to segment recordings into discrete blocks. Each approach carries distinct methodological and practical implications for data quality, system synchronisation, and subsequent analysis.

A continuous recording paradigm offers the advantage of preserving the natural flow of interaction without introducing artificial breaks that might alter participant behaviour. This approach minimises the risk of missing transient events, ensures that contextual information across modalities is retained, and simplifies synchronisation because all data streams are maintained within a single temporal frame. However, continuous recordings also present challenges. The resulting datasets are typically large, increasing demands on data storage, bandwidth, and system stability. Furthermore, long recordings elevate the risk that a hardware or software error—such as a dropped frame, sensor drift, or buffer overflow—can compromise extended portions of data rather than a single trial.

In contrast, segmented recordings divide the session into shorter blocks that can be aligned with experimental conditions or behavioural markers of interest. This design facilitates real-time quality checks after each segment and allows problematic segments to be repeated without affecting the entire dataset. Segmentation also reduces file sizes, making data management, transfer, and processing more efficient. However, frequent interruptions may influence the ecological validity of the interaction, as participants become more aware of the experimental structure. Moreover, synchronisation across systems must be carefully planned, as re-initialisation at the start of each segment may introduce variable latencies or time-stamp inconsistencies.

Ultimately, the choice between continuous and segmented recording should be guided by the research question, tolerance for system risk, and the priority placed on ecological validity versus experimental control. In practice, hybrid approaches may be optimal; for example, running long continuous recordings while embedding markers that allow post-hoc segmentation during analysis. Such strategies balance the robustness of continuous monitoring with the flexibility and manageability of segmented data streams.

In our implementation, a hybrid acquisition strategy was adopted. Recordings were run continuously within each experimental block to preserve the natural flow of interaction, while explicit temporal trigger markers were embedded to enable post-hoc segmentation aligned with stimulus onset, offset, and behavioural events. This approach minimised interruptions to participant interaction while maintaining flexibility for quality control and condition-specific analysis.

### Recording Protocol

**Figure 5.**
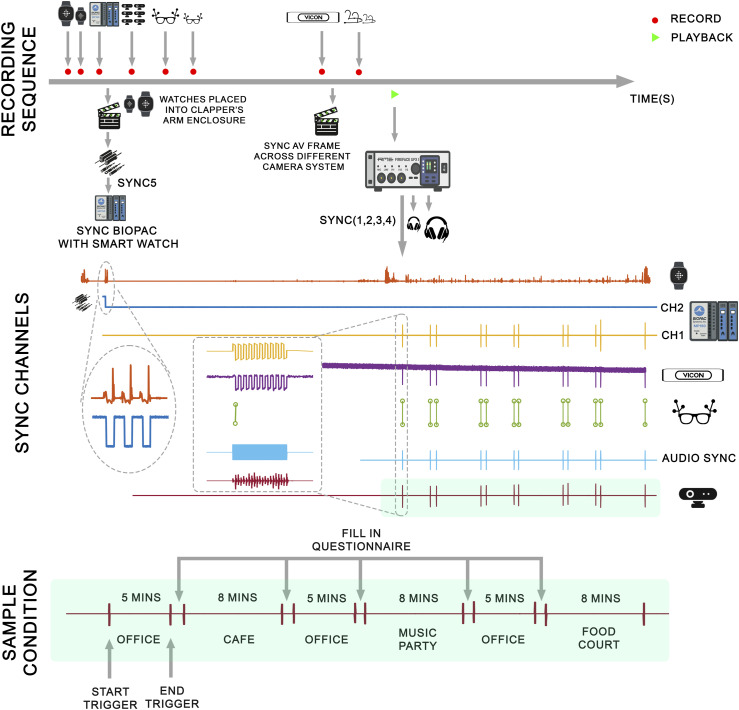
Recording sequence and synchronisation protocol across the multi-sensor acquisition system. Red circles denote the onset of recording for individual devices and streams in the physiological (smartwatch, BIOPAC), behavioural (gesture-tracking webcams, eye-tracking glasses, Vicon), and audio (DPA microphones) subsystems, whereas the green triangle marks the onset of noise playback. A clapper-generated photosensor signal (SYNC5) was used to synchronise the physiological subsystem, specifically the smartwatch and BIOPAC recordings (dotted ellipse). Three claps (arm open - close) motion was performed for every synchronisation event. RME trigger signals (SYNC1–4) were sent and received at the remaining subsystems to mark the onset and offset of each noise-playback condition (dotted rectangle). Additional clap-based synchronisation aligned audio and video across camera systems. An example condition is shown to illustrate the timing of synchronisation markers relative to the task sequence

A key component of our hybrid recording approach was to establish a clear recording protocol. The protocol allowed the experimenter to reliably navigate the complex steps and processes required to set up the recording system and ensured system stability, reliable synchronisation, and recoverability in the event of partial subsystem failure. Given the distributed acquisition architecture and heterogeneous timing requirements across sensors, systems had to be initialised in a fixed sequence as summarised in Figure 5.

Systems that needed to be calibrated individually were prepared prior to participant arrival to establish stable spatial and temporal reference frames. Motion-capture calibration was performed to define a consistent three-dimensional coordinate system for subsequent tracking. Wearable devices were then checked for battery capacity and storage availability, and their internal clocks were manually aligned with the laboratory reference time to support later temporal alignment.

Physiological recording systems were initiated early in the protocol to ensure uninterrupted acquisition of baseline and task-related signals. Synchronisation between wearable and laboratory-based physiological streams was achieved using the light-trigger mechanism placed behind the clapper’s arm, providing a precise temporal reference independent of software-based timing.

Participant-facing systems were prepared only after backend acquisition systems were confirmed to be stable. Video-based gesture recording and eye-tracking systems were initiated following participant arrival, with eye-tracking calibration performed immediately prior to recording to maximise accuracy. Motion capture and speech recording were then activated to ensure alignment with subsequent stimulus triggers.

To support recoverability across modalities, redundant multi-sensor timing cues were embedded prior to stimulus presentation. Using the purpose-built clapper, salient audiovisual and kinematic events were created to provide fallback reference points for post-hoc alignment in cases where primary hardware triggers were not captured by all systems.

Stimulus presentation was initiated last, with hardware triggers distributed simultaneously to all acquisition systems. This sequence ensured that all data streams were actively recording prior to stimulus onset, establishing a shared temporal reference for multi-sensor analysis.

In complex multi-sensor recording environments, partial system failures or missed synchronisation events are sometimes unavoidable. A pragmatic strategy is to assess, at the time of recording, whether sufficient redundant timing cues are available to support reliable post-hoc alignment. When clear audiovisual or kinematic markers are present, acquisition may proceed and alignment recovered during analysis. In contrast, when both primary triggers and redundant markers are absent for a given subsystem, experimenters should make a study-specific decision about whether to repeat the affected block, balancing the need to preserve technical interpretability against the potential for repetition to alter participant experience, task familiarity, or interactional behaviour. Defining such decision rules in advance supports consistent data quality while minimising unnecessary data loss.

### Data Storage

Multi-sensor recordings generate high-volume, heterogeneous data streams that are impractical to manage reliably using local storage alone, particularly when datasets need to be accessed by multiple researchers or preserved over extended project timelines. Sharepoint (Centralised cloud-based storage) was therefore selected to support long-term data integrity, secure access, collaborative sharing, scalability and redundancy against local hardware failure.

Given the complexity and size of multi-sensor recordings, explicit data integrity verification was treated as a core design requirement rather than an implicit assumption. To ensure that files were not corrupted, truncated, or altered during transfer or storage, checksum-based verification was implemented for all uploaded data. Cyclic redundancy check (CRC) comparisons allowed bit-level validation of files before and after transfer, providing assurance that recorded data remained identical across storage environments.

To minimise manual handling and the risk of human error, the open-source software rclone was used to automate the upload process and to verify post-transfer data integrity. After files were copied to SharePoint, rclone compared the local source files with the corresponding files stored remotely by checking file metadata such as file size and, where supported, checksum information. For Microsoft SharePoint storage, this checksum information includes Microsoft’s QuickXorHash, which provides a file-level hash that rclone can use to verify whether the uploaded file matches the original source file. Data were organised using a hierarchical storage structure in which each recording session formed the top-level directory, with nested subdirectories corresponding to individual acquisition systems, including BIOPAC, Tobii (S1), Tobii (S2), webcams, Nexus, audio, and metadata.

### Safety Measures

Safety considerations form a core design requirement for a multi-sensor laboratory given the integration of multiple powered devices, physiological sensors, and prolonged participant exposure to auditory stimulation. These unique electrical and comfort-related risks must be explicitly managed to protect both participants and research personnel.

To mitigate the risk of electrical power line leakage to sensors attached to participants, a medical-grade isolation transformer (Toroid ISB-060W) was incorporated into the experimental setup. All equipment was powered via this transformer, including the BIOPAC physiological recording system, which itself already provides galvanic decoupling, thus providing an additional layer of protection beyond standard laboratory power configurations.

Participant comfort and safety during auditory stimulation were also carefully addressed. Because realistic sound environments may reach uncomfortable levels for some individuals, the auditory presentation system incorporated a dedicated toggle button on the experimenter interface, which sent an OSC message to Max/MSP to reduce the output gain. This provided an instant, experimenter-controlled means of reducing playback levels in response to participant discomfort without disrupting system operation or compromising data integrity.

Together, these measures reflect a safety-by-design approach in which electrical isolation and real-time stimulus control are integrated into the system architecture rather than treated as procedural safeguards alone.

## Example Experiment

Below, we briefly describe an example experiment to illustrate how the technologies and methods of the proposed multi-sensory laboratory can be deployed to investigate interactive communication under realistic listening conditions. The experiment follows the recording sequence shown in Figure 5, with the research background, motivation, and outcomes described in Luthy et al. (2026).

A pseudo-randomised presentation order was used, with a consistent office-noise reference condition included to allow autonomic activity to return toward baseline following potentially arousing noise exposure. To minimise startle responses and abrupt physiological transients, participants were first exposed to a moderate, everyday environment (café or food court), while the music party condition—the highest-intensity stimulus—was always presented later.

Following each test condition, participants completed a brief questionnaire assessing their perceived communication experience in the given auditory environment. This design enabled comparison between subjective appraisal and concurrently recorded physiological and behavioural measures across listening conditions.

**Figure 6.**
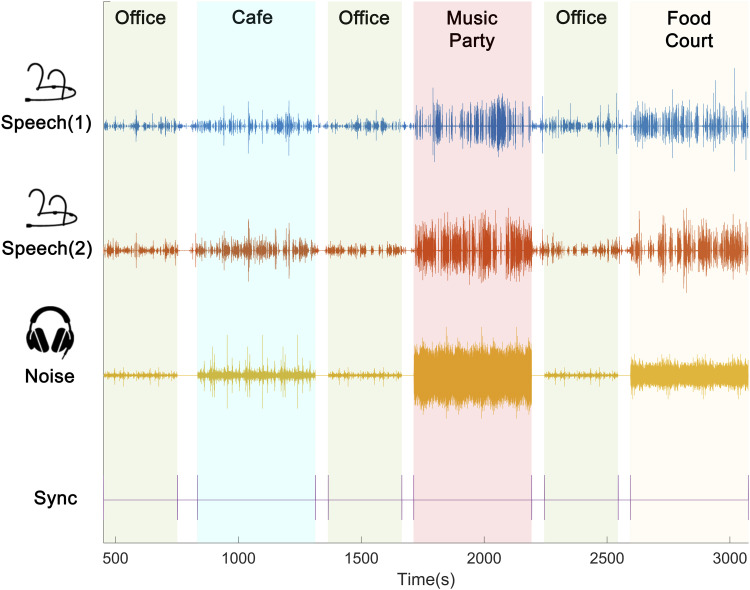
Representative time-aligned acoustic recordings from one experimental session. The first and second traces show the speech signals recorded from Participant 1 and Participant 2, respectively. The third trace shows the background noise associated with each acoustic scene, displayed here as a mixed noise trace for visual clarity rather than as separate multi-channel noise streams. The fourth trace shows the synchronisation signal, with event markers indicating the onset and offset of acoustic-scene playback. Coloured vertical bands indicate the sequence of realistic acoustic scenes presented during the session: Office, Café, Office, Music Party, Office, and Food Court

The audio recordings of the example dataset from one experimental session is presented in Figure 6. The acoustic scenes were presented in the following order: Office, Café, Office, Music Party, Office, and Food Court. The first and second traces show the speech signals recorded from Participant 1 and Participant 2, respectively, while the third trace shows the corresponding background noise across the different scenes. For visual clarity, a down-mixed (single-channel) version of the background noise is displayed instead of separate multi-channel noise streams.

The coloured vertical bands indicate the timing and identity of each realistic acoustic scene. The speech waveforms show that both participants produced speech throughout the session, with variations in speech activity across the different listening environments. Visual inspection also suggests an increase in vocal level during louder acoustic scenes, particularly during the Music Party and Food Court conditions, consistent with a compensatory increase in vocal output under increased background noise (Bottalico et al., 2022). The fourth trace shows the synchronisation signal, with event markers indicating the onset and offset of each acoustic scene playback. Together, these traces provide an overview of the recorded speech, background noise, and synchronisation structure used to align the acoustic and experimental events within the session.

**Figure 7.**
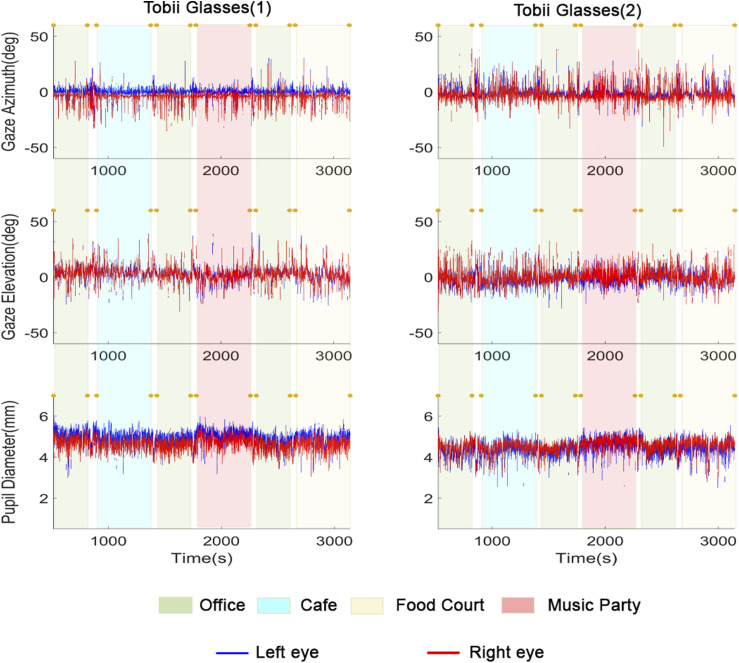
Representative visual behavioural recordings and Tobii Glasses eye-tracking data from one experimental session. These figures show time-aligned eye-tracking data from Participant 2 on the left and Participant 1 on the right. Gaze azimuth is shown in the top row, gaze elevation in the middle row, and pupil diameter in the bottom row. Blue and red traces represent the left and right eye. Shaded vertical bands indicate the acoustic scenes presented during the session, including Office, Café, Food Court, and Music Party conditions. Dots above the traces indicate synchronisation/event markers used to align the eye-tracking recordings with the experimental timeline

Figure 7 presents the corresponding time-aligned visual behavioural data captured during the experimental session. These figures show time-aligned Tobii Glasses recordings, with data from Participant 2 presented on the left and data from Participant 1 presented on the right. For each participant, the top row shows gaze azimuth, the middle row shows gaze elevation, and the bottom row shows pupil diameter. Blue and red traces indicate the left and right eye, respectively.

The shaded vertical bands denote the acoustic scenes presented during the session in line with the audio recordings from Figure 6. The dots above the traces indicate synchronisation or event markers used to align the eye-tracking recordings with the experimental timeline. Across the session, gaze azimuth and elevation values were generally centred around zero degrees, indicating that participants predominantly oriented their gaze toward the communication partner positioned in front of them.

**Figure 8.**
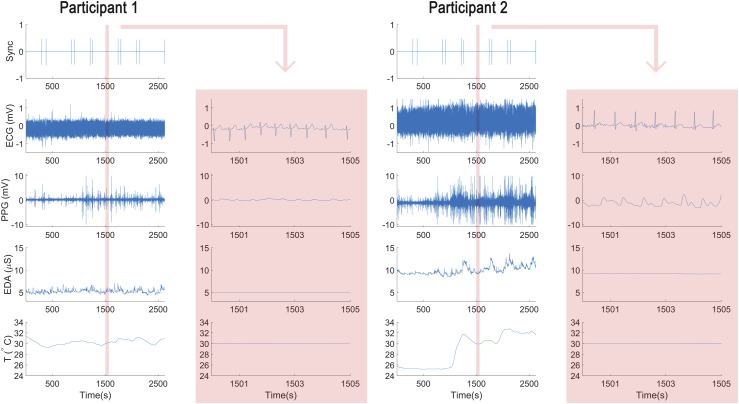
Representative synchronised Physiological Sensing Subsystem recordings from two participants during a sample experimental session. Signals include ECG recorded from electrodes placed on the chest, and PPG, temperature, and skin conductance recorded from sensors attached to the fingers of the non-dominant hand. The first and third columns show the full-session recordings for Participant 1 and Participant 2, respectively, whereas the second and fourth columns show corresponding 5-s segments from each participant’s data

Figure 8 shows the corresponding, time-aligned physiological recordings obtained from the two participants during the experimental session. Multiple autonomic nervous system measures were recorded simultaneously, including ECG from electrodes placed on the chest, and PPG, electrodermal activity, and skin temperature from sensors attached to the fingers of the non-dominant hand. The full-session traces confirm that all physiological streams were recorded continuously throughout the experimental session for both participants.

The synchronisation channel shows clearly defined event markers throughout the recording, indicating that timing information was successfully captured alongside the physiological signals. The 5-s segments shown in the red boxes for each participant provide a closer view of the physiological waveforms around a selected synchronisation point. In these expanded segments, the ECG signal shows identifiable cardiac cycles, while the PPG signal exhibits slower pulse-related fluctuations. EDA and temperature vary more gradually over time, as expected for measures reflecting slower autonomic and peripheral physiological changes.

Differences in signal characteristics were observed between the two participants. Participant 2 showed larger variation in the PPG and EDA traces across the session compared with Participant 1, while temperature also showed a more pronounced change over time. In contrast, Participant 1 showed relatively stable EDA and temperature traces across the recording period. Together, these data demonstrate that the system was able to capture synchronised, multi-channel physiological responses from both members of the dyad, providing time-aligned measures suitable for subsequent analysis of autonomic activity during interactive communication.

## Lessons Learned and Recommendations

Developing and operating a complex multi-sensor communication laboratory highlighted several aspects critical for robustness and reproducibility. Foremost, explicitly defining subsystem requirements prior to integration proved essential. Treating each sensing modality as an independent yet temporally coordinated component helped clarify interface requirements, reduce integration errors, and streamline system expansion.

Simplicity emerged as a key design principle. Minimising unnecessary dependencies and avoiding over-centralisation reduced the number of potential failure points and improved system stability during extended recordings. In particular, distributed acquisition with clearly defined synchronisation interfaces proved resilient.

Reliable temporal alignment required synchronisation to be treated as a first-order design principle rather than a post hoc processing step. Planning explicit synchronisation pathways in advance—along with redundant fallback markers—substantially reduced the risk of irrecoverable timing errors when devices operated at different sampling rates or experienced intermittent trigger loss.

Operational experience also underscored the importance of real-time feedback and immediate post-session validation. Providing experimenters with live indicators of system status enabled early detection of anomalies, while systematic inspection of recorded data immediately after acquisition allowed issues to be identified before subsequent sessions. Finally, incorporating redundancy and backup pathways across acquisition, synchronisation, and storage proved critical for mitigating the impact of hardware failures or transient software issues in the high-bandwidth multi-sensor recordings. However, it should be noted that falling back to a secondary synchronisation measure always results in significant effort during post-processing, often involving time-consuming manual processes.

If there is one overarching lesson from building and operating this laboratory, it is that participant safety must be treated as a first-order design consideration rather than a late-stage compliance requirement. In our system, the inclusion of a medical-grade isolation transformer was added after recognising the potential risks associated with combining mains-powered physiological recording equipment with direct participant contact. This experience highlighted the need to consider electrical isolation from the outset, before other integration decisions are finalised. We strongly recommend that groups building similar systems seek appropriate technical and safety advice early in the design process and prioritise electrical isolation as a core architectural requirement.

Synchronisation was more difficult than anticipated. The challenge was not any single device or connection, but the combination of heterogeneous sampling rates, differing trigger voltage requirements, and the simple reality that systems designed for lower-rate data acquisition may miss fast trigger pulses. Through iterative testing, extensive piloting, and the deliberate introduction of redundant synchronisation pathways, we were able to substantially reduce the risk of synchronisation failure. Any group implementing a similar system should expect synchronisation to require substantial development time and should build, test, and document redundancy before collecting experimental data.

Reflective surfaces in the experimental room presented an unexpected challenge for motion capture performance. During system development, spurious reflections from room furnishings — specifically the coffee table and the backs of participant chairs — produced erroneous marker detections that degraded tracking quality and, in some cases, rendered data unusable. Resolving this issue required systematic comparison of tracking performance across different room configurations, ultimately necessitating both camera repositioning and the removal or covering of reflective surfaces. We recommend that researchers implementing motion capture systems conduct a thorough assessment of all reflective surfaces within the capture volume, including furniture, flooring, and equipment casings, before finalising camera placement.

Providing experimenters with real-time indicators of system status enabled early detection of acquisition anomalies during recording sessions. Equally important was the systematic inspection of recorded data immediately following each session, which allowed issues to be identified and addressed before subsequent recordings were conducted. While manual inspection of recorded files is feasible, it is time-consuming and prone to oversight, particularly given the volume and heterogeneity of data streams generated in multi-sensor recordings. We therefore recommend developing automated post-session verification scripts that systematically iterate through acquisition folders and flag missing files, unexpected file sizes, and potential data integrity issues. Implementing such automated checks as a routine component of the post-session workflow can substantially reduce the burden of quality control and increase the likelihood that data integrity issues are detected promptly, before they propagate across multiple recording sessions.

Instead of viewing the present system as a fixed template, we suggest that multi-sensor laboratory design be approached as a set of transferable principles that can be adapted to different sensor combinations, acquisition platforms, and research goals. Early specification of all subsystems — including sensors, acquisition hardware, software environments, and expected data streams — is critical, as sampling requirements, data bandwidth, storage demands, trigger compatibility, and API accessibility substantially constrain the architectural options available. Treating each modality as an independent but temporally coordinated subsystem can help clarify interface requirements, reduce integration errors, and support future system expansion. More broadly, our experience suggests that successful multi-sensor laboratory development depends not only on selecting appropriate technologies, but also on designing for safety, redundancy, verification, and adaptability from the outset.

The choice between a centralised and decentralised architecture should then be made explicitly rather than implicitly. A centralised architecture may simplify acquisition, synchronisation, and downstream processing by bringing multiple data streams into a common framework or middleware environment. However, such an approach depends on compatible devices, accessible APIs, sufficient bandwidth, and acceptable robustness as system complexity increases. Where these conditions are not met, a decentralised architecture may be more practical and resilient, particularly when involving several simultaneous high-bandwidth data streams. In our own case, the presence of multiple concurrent video recordings made a fully centralised solution impractical, and distributed acquisition with clearly defined synchronisation interfaces proved more robust.

The synchronisation strategy should likewise be selected in relation to the chosen architecture and the technical capabilities of the constituent subsystems. In centralised systems, synchronisation can often be handled directly within the shared acquisition environment. In decentralised systems, by contrast, investigators must determine how each subsystem can receive, record, or otherwise register temporal markers. Some research-grade devices provide dedicated auxiliary or trigger input channels that permit direct recording of synchronisation pulses. Other devices, particularly consumer-oriented platforms, may not offer such functionality and may therefore require indirect synchronisation approaches based on signals already available in the recorded data. In our setup, for example, the smartwatch did not provide any direct input for a dedicated trigger signal, and synchronisation was therefore achieved by attaching the smartwatch to a clapper board and detecting the impact when closing it through the accelerometer stream. Where fine-grained synchronisation is not essential, shared timestamps referenced to a common clock or time server may also provide a useful alternative for coarse temporal alignment.

An important general principle is to build redundancy into the synchronisation scheme wherever possible. Primary synchronisation pathways should be complemented by secondary or backup markers so that approximate alignment can still be reconstructed if the principal method fails. This is especially important in systems with heterogeneous sampling rates or occasional trigger loss. At the same time, fallback synchronisation should not be regarded as cost-free, as reliance on secondary markers can substantially increase post-processing effort and often requires time-consuming manual correction.

Operational considerations are equally important. A detailed test protocol is essential to guide the experimenter through the complex calibration and initialisation steps that need to be executed in strict temporal order to ensure data synchronisation and integrity. Having a technical expert on call will help to promptly resolve any unexpected problems occurring during testing, and thereby avoid re-booking of valuable test participants. Real-time monitoring of subsystem status facilitates early detection of acquisition problems, while immediate post-session inspection of recorded data can help identify failures before subsequent sessions are run. Redundancy should also extend beyond synchronisation to include acquisition and storage pathways, thereby reducing the impact of transient hardware or software failures. More generally, simplicity remains a valuable design principle: minimising unnecessary dependencies and avoiding over-centralisation can reduce potential points of failure and improve stability during extended recording sessions.
